# Structure, optical and magnetic properties of a novel homometallic coordination polymers: Experimental and Computational studies

**DOI:** 10.1038/s41598-020-58176-3

**Published:** 2020-01-28

**Authors:** Y. Ammari, N. Baaalla, E. K. Hlil, S. Abid

**Affiliations:** 1 Université de Carthage, Faculté des sciences de Bizerte, LR13ES08 Laboratoire de chimie des matériaux, 7021 Zarzouna Bizerte, Tunisie; 2 Institut Néel, CNRS, Université Grenoble Alpes, 25 rue des Martyrs BP 166 38042, Grenoble cedex, 9 France; 3 LaMCScI, Laboratory of Condensed Matter and Interdisciplinary Sciences, B.P. 1014, Faculty of Science, Mohammed V University, Rabat, Morocco

**Keywords:** Magnetic materials, Chemical physics, Coordination polymers

## Abstract

Single crystal of 1D homometallic coordination polymer involving cobalt metal ion and P_2_Mo_5_ Strandberg type polyoxometallate cluster (C_6_H_10_N_2_)_2_[Co(H_2_O)_4_P_2_Mo_5_O_23_].6H_2_O, is prepared in aqueous solution and characterized by X-ray diffraction (XRD), UV-vis diffuse reflectance, fluorescence and magnetism. Single crystal X-ray diffraction analysis reveals that this compound crystallizes in the triclinic system with space group P $\,\bar{1}$. The 3-(ammoniomethyl)pyridine C_6_H_8_N_2_ organic fragment is used merely as stabilizer for the promotion of topological structure. DRS data indicate that the synthesized material can be identified as a ferromagnetic semiconductor with optical bands gaps energy of 1.81 and 2.74 eV, respectively. The large value of refractive index observed in the visible region make the simple a promising candidate for visible optical communication devices and fluorescent emisson result provides that the complex belongs to a blue luminescent compounds. Moreover, magnetic measurements and electronic structure calculations show that P_2_Mo_5_ Strandberg polyoxoanion can be reported as a new class of ligand that is candidate to construct metal-inorganic framworks with long distance ferromagnetic superechange between Co(II) centers. The evidence from this study suggests that the syntesized polymer can become a great multifunctional material openning the door for the developpement of new coordination polymers based on Strandberg type polyxoxmetalate with potential applications.

## Introduction

Metal–inorganic hybrid frameworks are one of the brilliant candidates of multifunctional materials owing to their interesting topological structures and many potential behaviours and applications^1–3^. Among the versatile characteristics of multifunctional materials; structure, optical and magnetic properties accurately represent a huge challenge for the scientists of modern technology owing to their encouraging applications such as spintronics, high-density information storage and solar cell conversation^4,5^. Although, this ambition can be systematically generated by selecting the suitable starting reagents (metals ions, inorganic building units and organic groups).

As a fascinated class of inorganic metal-oxygen building units, Strandberg- type polyoxometalate P_2_Mo_5_ appears to be a dominant class of metal-oxo cluster anions that exhibit numerous remarquable behaviors, such as high electron density, good stability and especially ligand to metal charge transfer (LMCT) optic phenomena^6,7^. Furthermore, the incorporation of a paramagnetic transition metal with P_2_Mo_5_ polyoxoanion cluster open the door to explore new chemistry paths in fabricating heterostructures and ultracompact devices with magnetic and optical properties.

In the last few years, several P_2_Mo_5_ complexes including discrete cluster, 1D chains, 2D layered structures and 3D frameworks have been reported^8–10^. For instance, J. Thomas groups reported a series of copper complexes based on the P_2_Mo_5_ clusters and indicated the influence of pH and temperature in the crystallinity of the synthesized compounds^11^. In 2014, Z.You and al. synthetized a three new molybdophosphate complexes and revealed their Curie–Weiss paramagnetic behaviour^12^. Recently,we have successfully isolated a new inorganic-organic hybrid compound basrd on strandberg-type phosphomolybdate and copper cation and investigated the antiferromagnetic interactions between the copper centers^13^. However, some optical electronic properties like refractive index, dielectric constant and electronic structure calculation of these compounds families have not been investigated up to now. Thus, based on aformentioned consideration, we successfully isolated a multifunctional molecular material based on Strandberg-type P_2_Mo_5_ cluster and cobalt cation Co(II). Even so, 3-(ammoniomethyl)pyridine organic fragment is used merely as stabilizer for the promotion of topological structural diversification. Additionally, X-ray diffraction, optical, magnetic and electronic structure calculations are carried as well.

## Methods

### Synthesis of the sample

All chemical elements were commercially purchased and used without further purification.

A 50 mL of an aqueous solution containing ammonium heptamolybdate (NH_4_)_6_[Mo_7_O_24_].4H_2_O (0.952 g – 0.77 mmol) is slowly added to an aqueous solution of cobalt (II) chloride CoCl_2_.2H_2_O (0.166 g – 1 mmol) and 3- picolylamine (0.216 g − 2 mmol). Experimental results indicate that reasonable yields (49%) of crystalline products can be obtained when the pH value of the mixture is adjusted to 3–4 with 85% H_3_PO_4_ under continuous stirring. Two weeks after, purple crystals that are suitable for single crystal X-ray diffraction were obtained. Elemental analysis for C_12_CoH_42_Mo_5_N_4_O_33_P_2_ (1371.07) leads to calculated (wt %): C, 10.50; H, 3.06; N, 4.08 found (wt %): C, 10.49: H, 3; N, 4.10

### X-ray diffraction and measurements of physical properties

Intensity data were collected on a Nonius Kappa CCD diffractometer with graphite-monochromated $Mo{K}_{\bar{\alpha }}$ (*λ* = 0.71073 Å) radiation at room temperature. The crystal structure is solved by direct method and refined by full-matrix least-squares on F^2^ using the SHELXTL-97 program^14^. Anisotropic thermal parameters were used to refine all non-hydrogen atoms. The H atoms were placed geometrically and refined using a riding model. In Addition, the Molecular Hirshfeld surfaces are generated by CrystalExplorer computer program^15^.

The crystallographic data and refinement details are summarized in Table 1. Selected bond lengths, bond angles and the magnitude of distortion are listed in Table S1. Bond distances and angles of H-bond network are given in Table S2.

**Table 1:** Crystal Data, Measurement Parameters, and Structural Refinement Parameters of (C_6_H_10_N_2_)_2_[Co(H_2_O)_4_P_2_Mo_5_O_23_].6H_2_O.(CCDC:1576104).

Empirical formula	C_12_H_42_CoMo_5_N_4_O_33_P_2_
Fw (g.mol^−1^)	1371.07
Crystal system	triclinic
Space group	P$\bar{1}$
a(Å)	11.1205 (5)
b(Å)	11.7576 (5)
c(Å)	16.7869 (8)
α (°)	80.761 (4)
β (°)	83.937 (4)
γ (°)	62.411 (4)
Volume(Å^3^)	1918.86 (15)
Z	2
Density (g.cm^−3^)	2.373
Wavelength	0.71073 Å (Mo $({K}_{\bar{\alpha }}))$
Theta min-max(°)	3–25.5°
F(000)	1346
Data/restraints/params	16524/1/540
Independent reflections (I > 2σ(I))	8919 (7541)
Goodness of fit	1.07
R, wR_2_,	0.031, 0.078
Δρ_min_, Δρ_max_ (eÅ^−3^)	−1.18, 0.64

For spectral measurements, the UV–Vis diffuse reflectance is performed on a Perkin–Elmer spectrophotometer type instrument Lambda-45 coupled to an integration sphere type RSA-PE-20 in the range of 200–700 nm with a speed of 960 nm. min^−1^ and an aperture of 4 nm. Infrared (IR) spectrum was recorded at a room temperature on a Nicolet IR200 FTIR spectrophotometer in the 4000–400 cm^−1^ region. Excitation and emission spectra were measured with Perkin–Elmer LS55 Fluorimeter using solid samples at room temperature.

Magnetic measurements are performed as well. The temperature dependence of the magnetization was carried out using a BS_2_ magnetometer developed in Louis Neel Laboratory of Grenoble with an external magnetic field equal to 500 Oe in the temperature range 2–330 K. Magnetization measurements M (μ_0_H) were performed with variable field μ_0_H up to 10^5^ Oe at 2 K.

### Electronic structure calculations

We used the Full Augmented Plane Wave (FLAPW) method^16^ which performs DFT calculations with the generalized gradient approximation (GGA). The Kohn-Sham equation and energy functional were evaluated consistently. For this doing, the space was divided into the interstitial and the non-overlapping muffin-tin spheres centered on the atomic sites. The basis function inside each atomic sphere consisted in linear expansion of the radial solution of a spherically potential multiplied by spherical harmonics. In the interstitial region, the wave function was taken as an expansion of plane waves and no shape approximation for the potential was introduced in this region consistently with the full potential method. The core electrons were described by atomic wave functions solved relativistically using the current spherical part. Spin polarized potential as well as the ferromagnetic states are considered. The atomic muffin-tin (MT) spheres, supposed not to overlap with each other, are taken as 1.80, 2.10, 1.64, 1.04, 1.12 and 0.55 a.u for Co, Mo, P, N, C and H atoms, respectively. The gap energy, which defines the separation of the valence and core state, was chosen equal to −6.0 Ry. The largest reciprocal vector G in the charge Fourier expansion, G_max_, was equal to 28 and the cut-off energy corresponding to the product of the muffin-tin radius and the maximum reciprocal space vector (RMT. K_max_) was equal to 7. Inside the atomics spheres, the potential and charge density are expanded in crystal harmonics up to l_max_ = 6. Calculations are performed with 16 inequivalent k-points in the irreducible Brillouin zone. Such a value is large enough to ensure both the gap and the magnetic moment. The convergence criterion was chosen to be the total energy and set at 10^−4^ eV. For computation, our refined XRD lattice parameters are used.

## Results and Discussion

### Crystal structure

The experimental powder X-ray diffraction pattern of the title compound is revealed in good agreement with the simulated pattern derived from the model coordinates, indicating the good phase purity of the sample **[**Fig. S1**]**. The difference in intensity is due to the preferred orientation of the crystalline powder samples.

**Figure 1:**
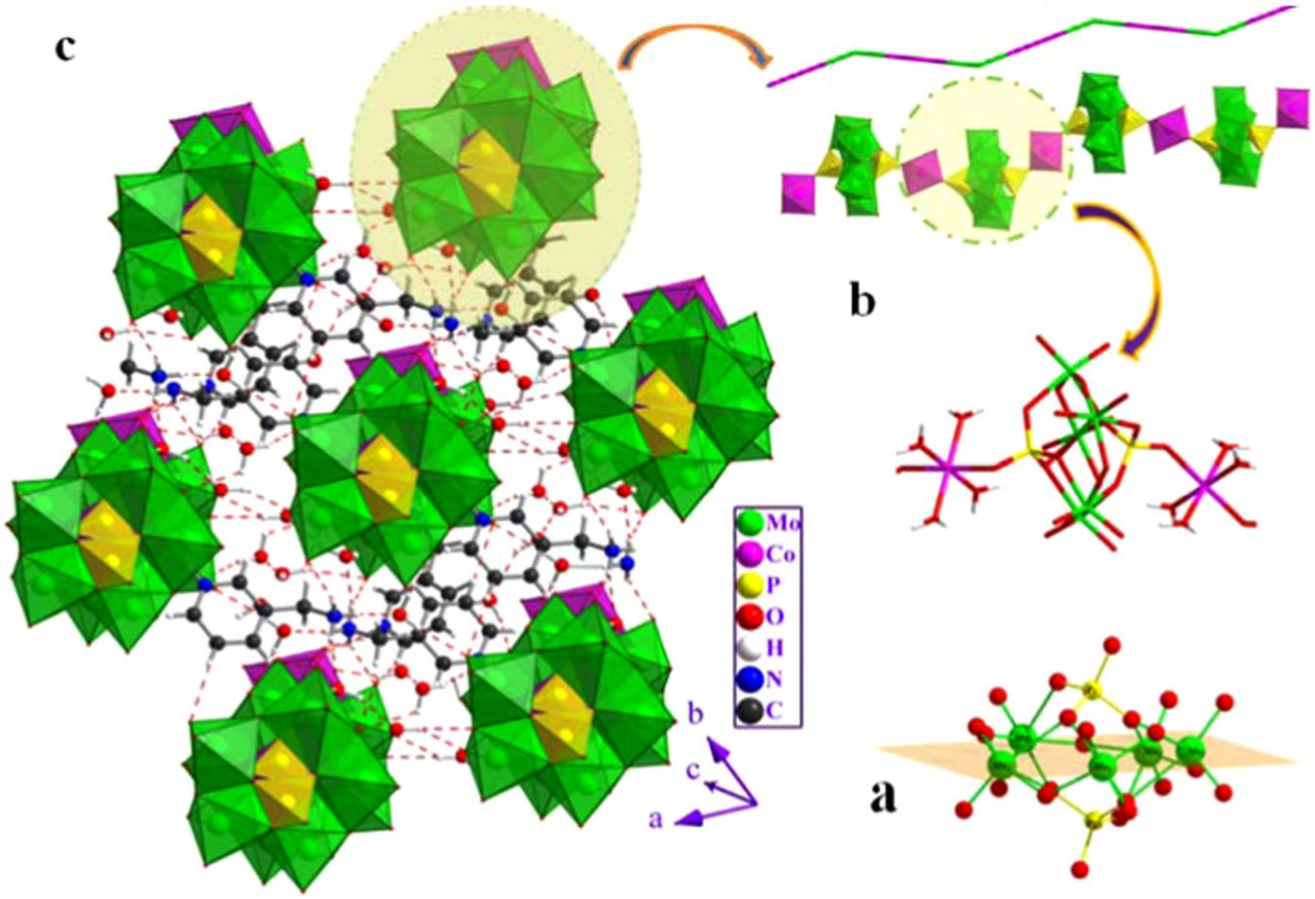
(**a**) Polyhedral and ball and stick representation of the 3-D structure of (C_6_H_10_N_2_)_2_[Co(H_2_O)_4_P_2_Mo_5_O_23_]- .6H_2_O. The dashed lines denote the hydrogen interactions. (**b**) View of the 1-D chain of {P_2_Mo_5_} units linked by Co cations along the c axis in the compound; (**c**) Structural representation of the coordination environment of two Co(II) ions around the Strandberg-type {P_2_Mo_5_} cluster in the complex.

Crystal data indicates that (C_6_H_10_N_2_)_2_[Co(H_2_O)_4_P_2_Mo_5_O_23_].6H_2_O crystallizes in the centrosymmetric space group P $\bar{1}$, with one [P_2_Mo_5_O_23_]^6−^ polyanion,two half of Co^2+^ cations, two protonated 3-(Aminomethyl)pyridine and ten crystal water molecules in the asymmetric unit. Among these water molecules, two show disorder over two mutually exclusive positions with refined occupancies of 0.55(8): 0.45(8) and 0.44: (2) 0.56 (2), for OW_9_ and OW_10_, respectively. On the basis of bond valence sum calculations^17^, the oxidation states of P, Co and Mo atoms are  + 5, + 2 and  + 6, respectively. The Strandberg-type polyoxoanions [P_2_Mo_5_O_23_]^6−^ could be described as a nearly planer {Mo_5_} ring formed by five distorted edge- or corner-shared MoO_6_ octahedra, capped on either face by two PO_4_ tetrahedra in a corner sharing mode. Inside the Strandberg cluster, the Mo-O distances are in the range of 1.695–1.726 Å for terminal oxygen (Mo-Ot), 1.902–1.949 Å for (Mo-O_μ2_) and 2.190–2.392 Å for (Mo-O_μ3_), which can be distributed into three classes short bond, intermediate bond and long bond, respectively. All these distances are within the normal ranges^18,19^. The P atom exhibits a distorted tetrahedral geometry with P-O distances ranging from 1.512(3) to 1.553(2) Å, the O-P-O bond angles are in the range of 106.87(13)-110.78(14)° and the P-P distance is 3.795 Å. These distances and angles are in good agreement with observed in previously reported literatures^20^. The molybdenum atoms of the pentagonal ring are almost on the same plane with an rms deviation of 0.1615 Å **[**Fig. 1a**]**. The five MoO_6_ octahedra are distorted along the local C_2_[110] direction and the magnitude of the distorsion (Δd) quantified using the method proposed by Halasyamani^21^. The magnitude of the out-of-center distortion (Δd), ranging from 1.192 to 1.265 [Table S1], indicate a strong distortion type of MoO_6_ octahedra. The two crystallographically independent cobalt cations, located upon inversion centers, exhibit the same octahedral coordination environment made by four water molecules and two bridging oxo-groups from two adjacent {P_2_Mo_5_} clusters through the terminal oxygen atom of the PO_4_ tetrahedron. The Co-O bond lengths and O-Co-O bond angles, ranging from 2.047(2) to 2.127(3)Å and 83.9(11) to 96.1(11)°, respectively are consistent with the normal ranges observed in other complexes^22,23^. As seen in Fig. 1b, adjacent {P_2_Mo_5_} anions are connected to the Co(1) and Co(2) octahedron through the terminal oxygen atoms of two opposite PO_4_ tetrahedra to generate an infinite 1D zigzag chain structure along the [101] crystallographic direction. The distance between adjacent P_2_Mo_5_ clusters is 9.566 Å and the shortest intrachain Co⋯Co distance is close to 9.556 Å. The protonated 3-(ammoniomethyl)pyridinium dication and the uncoordinated water molecules, located in the void spaces, connect adjacent chains into a 3D supramolecular framework **[**Fig. 1c**]** through N-H…H(O, OW) and OW-H…(O, OW) hydrogen bonds **[**Table S2**]**. In addition, weak hydrogen contacts are present involving the carbon atom as H-donor and the oxygen atoms of the phosphomolybdate anions acting as an H-acceptor. The bond lenghs and angles of the (C_6_H_10_N_2_)^2+^ groups ranging, respectively, from 1.323(5)–1.514(6) and 112.1(4)–123.1(4), are within normal ranges^24^.

### Study of non-covalent interactions

**Figure 2:**
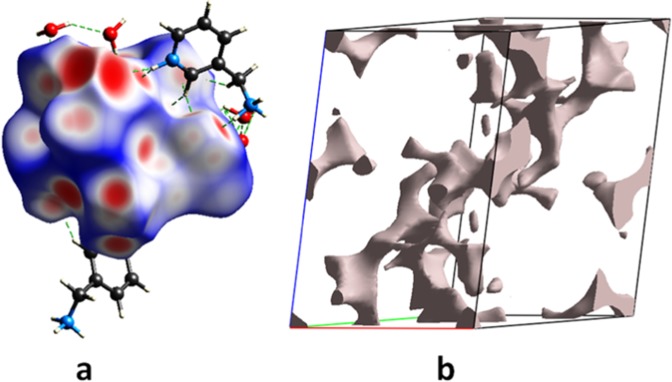
(**a**) Hirshfeld surfaces mapped at d_norm_ of (C_6_H_10_N_2_)_2_[Co(H_2_O)_4_P_2_Mo_5_O_23_].6H_2_O; (**b**) Voids of the crystal at the 0.002 a.u. isosurface.

Using Crystal Explorer 3.1, the supramolecular interactions between [Co(H_2_O)_4_P_2_Mo_5_O_23_]^4−^ anion, organic cations and water molecules are further analyzed by studying the Hirshfeld surface (d_norm_) and 2D fingerprint plots. Hirshfeld d_norm_ surfaces for the title complex have been mapped by using a red-blue color scheme in Fig. 2; where deep red areas assigned to closer contacts with d_norm_ = −0.818 Å, the blue regions correspond to longer contacts (d_norm_ = 1.322 Å) and the white regions indicate medium contacts (d_norm_ = 0.303 Å) which are contributed by the other intermolecular contacts. The interactions between the oxygen of {P_2_Mo_5_} cluster and the H atoms bounded to N and OW are shown as deep red areas in the Hirshfeld surfaces. The fingerplots of the complex show that H^…^O interactions (C-H…O, O-H…O and N-H…O) and H…H are dominate **[**Fig. S2a**]**. The contributions of the well defined are respectively 52.2% and 31.8% among all interactions. The relative contributions to the Hirshfeld d_norm_ surface area for the other intermolecular contacts are illustrated in Fig. S2b.

In order to study the propensity of two chemical species (X and Y) to be in contact, we have calculated the Hirshfeld contact surfaces, derived random contact and enrichment ratios (E_XY_)^25^ for (C_6_H_10_N_2_)_2_[Co(H_2_O)_4_P_2_Mo_5_O_23_].6H_2_O are given in Table S3. It is interestingly to note that the value of E_XY_ is expected to be usually larger than unity for pairs of elements with a high propensity to form contacts in crystals, while pairs that have a tendency to avoid contacts are associated with E_XY_ values lower than unity. The proportion of surface contacts of chemical type on the molecular Hirshfeld surface highlighted that this compound has a large number of hydrogen and oxygen atoms on their surface (S_H_ = 60% and S_O_ = 32.55%). While (C, N and Co) atoms are rarely present at the molecular surface (S < 5%), decreasing the value of the random contacts (R < 5%). The list of the enrichment ratios reveals that the O…H; C…O, N…O and Co…O contacts are highly enriched which turn out to be favored in the crystal packing. The N…H contacts can be considered enriched (E_NH_ = 1) while the H…H contacts are slightly enriched for structure (E_HH_ = 0.88). On the other hand, the N…H and C…H contacts are impoverished with E_NH_ and E_CH_ ratio of 0.55 and 0.16, respectively. Similarly, the proportion of carbon atoms on the molecular surface (S_C_ = 4.65%) as well as the lower value of the random contact (R_CC_ = 0.21%) have further strengthened our conviction that π…π interactions tend to be disfavored in directing the packing of (C_6_H_10_N_2_)_2_[Co(H_2_O)_4_P_2_Mo_5_O_23_].6H_2_O.

### Optical properties

**Figure 3:**
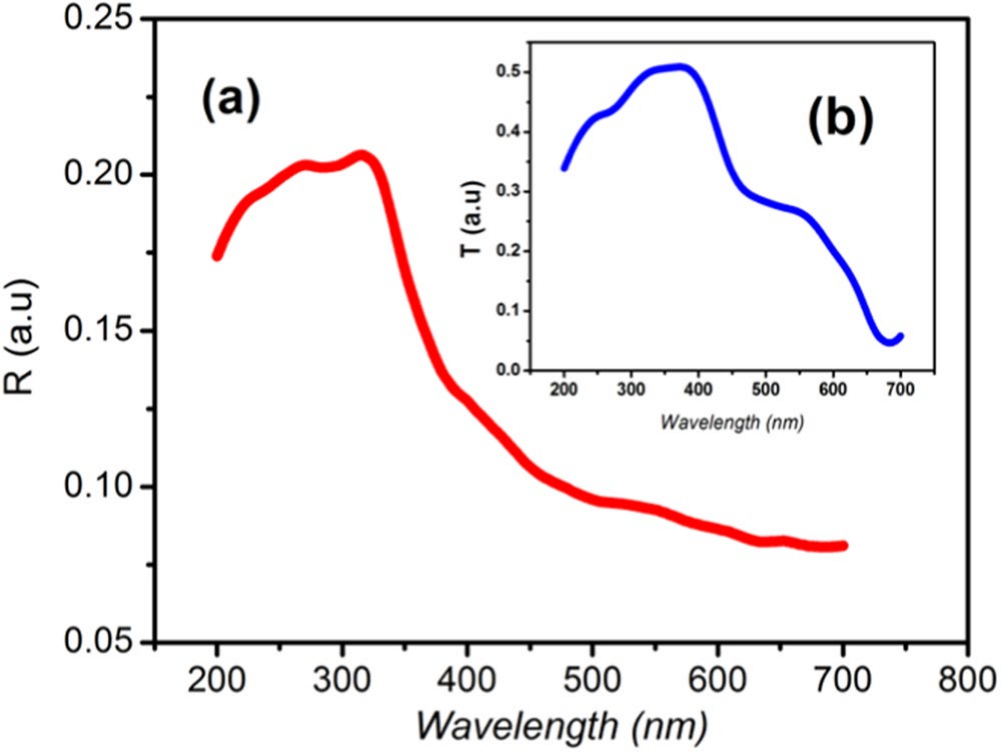
(**a**) Variation of the transmittance and reflectance with wavelength for (C_6_H_10_N_2_)_2_[Co(H2O)_4_P_2_Mo_5_O_23_].6H_2_O; (**b**) Variation of reflectance with transmittance.

The optical absorption behavior and band gap energy of (C_6_H_10_N_2_)_2_[Co(H_2_O)_4_P_2_Mo_5_O_23_].6H_2_O was studied by means of diffuse reflectance spectroscopy (DRS), as shown in Fig. 3a. The spectrum shows a maximum reflectance (~20%) in the region between 200 nm and 350 nm corresponding to lower absorption. From 400 nm, a decrease in reflectance is seen due to fundamental absorption (valance band to conduction band) by the material^26–28^. The transmittance spectrum indicates that the transmission coefficient varies between 5% and 50% at the UV-visible region which means that the studied compound is translucent in the studied wavelength region^29^ [Fig. 3b**]**.

**Figure 4:**
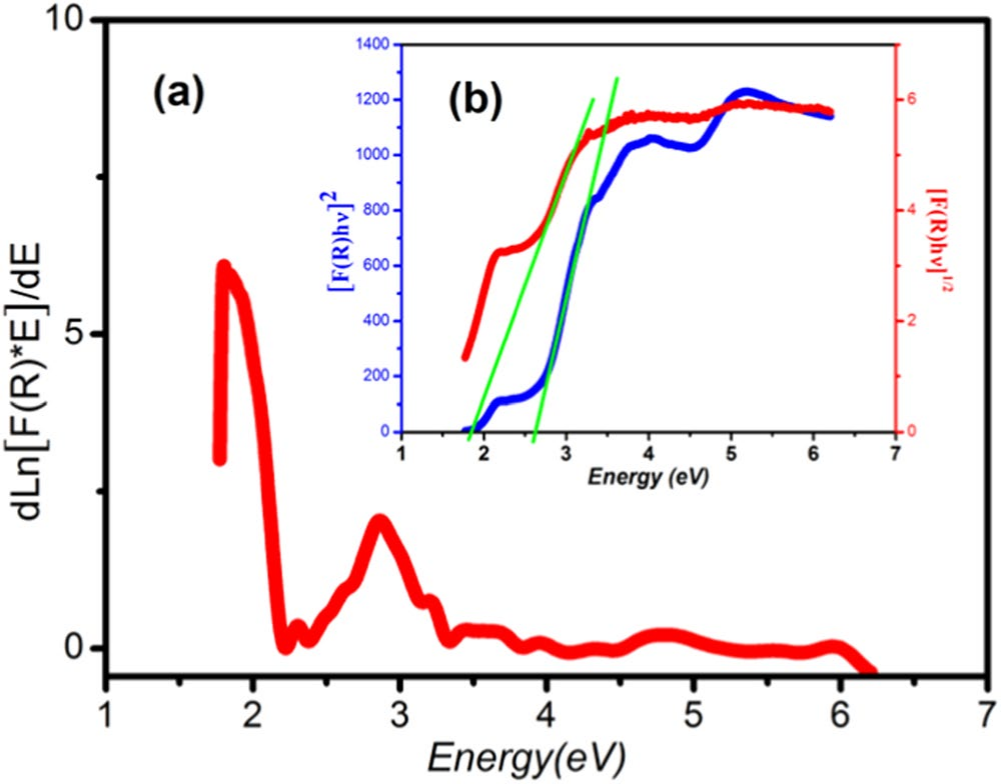
(**a**) The plot of $\frac{{\bf{d}}\,{\bf{L}}{\bf{n}}\,[{\bf{F}}({\bf{R}}){\bf{h}}{\boldsymbol{\gamma }}]}{{\bf{d}}\,({\bf{h}}{\boldsymbol{\gamma }})}$ versus Energy for the simple. (**b**) The plots of (F(R)hν)^2^ and (F(R)hν)^1/2^ as a function of photon energy (hν) for (C_6_H_10_N_2_)_2_[Co(H_2_O)_4_P_2_Mo_5_O_23_].6H_2_O.

The acquired diffuse reflectance spectrum is converted to Kubelka-Munk function ${\rm{F}}({\rm{R}})=\frac{{(1-{\bf{R}})}^{2}}{2{\bf{R}}}$, where R is the reflectance, which is proportional to the absorption coefficient α. The energy band gap of the sample is calculated using the relational expression proposed by Tauc, Davis, and Mott^30^. In the region of high absorption, E_g_ is connected to the absorption coefficient α of the following equation^31^: (F(R)hγ)^1/r^ = B(hγ – Eg), where B is constant, E_g_ is the optical band gap, h*υ* is the energy of the incident photon and (r) is an index which may be equal to ½ and 2 assigned to the allowed direct and allowed indirect transition, respectively. In the actual experiment, the α in the Tauc equation is substituted with F(R) and the relational expression becomes: (F(R)hγ)^1/r^ = B(hγ – Eg), In order to determine the value of Eg for the samples, we take the natural logarithm and first order derivation of the Tauc’s equation to get: $\frac{{\bf{d}}\,{\bf{L}}{\bf{n}}[{\bf{F}}({\bf{R}}){\bf{h}}{\boldsymbol{\gamma }}]}{{\bf{d}}\,({\bf{h}}{\boldsymbol{\gamma }})}=\frac{{\bf{r}}}{({\bf{h}}{\boldsymbol{\gamma }}-{{\bf{E}}}_{{\bf{g}}})}$ According to this equation a peak should appear in the curve of $\frac{{\bf{d}}\,{\bf{L}}{\bf{n}}\,[{\bf{F}}({\bf{R}}){\bf{h}}{\boldsymbol{\gamma }}]}{{\bf{d}}\,({\bf{h}}{\boldsymbol{\gamma }})}$ versus hν at the point where hν ≅ Eg. The plot of $\frac{{\bf{d}}\,{\bf{L}}{\bf{n}}\,[{\bf{F}}({\bf{R}}){\bf{h}}{\boldsymbol{\gamma }}]}{{\bf{d}}\,({\bf{h}}{\boldsymbol{\gamma }})}$ versus hν in the Fig. 4a shows that the compound has two bands gaps of 1.81 eV (~ 685 nm) and 2.74 eV (~ 460 nm), respectively. Shows the plots of (F(R)hν)^2^ and (F(R)hν)^1/2^ as a function of photon energy (hν) for (C_6_H_10_N_2_)_2_[Co(H_2_O)_4_P_2_Mo_5_O_23_].6H_2_O compound [Fig. 4b**]**, we can identify the first transition (1.81 eV) as allowed direct while the second (2.74 eV) as allowed indirect transition. Furthermore, the band tail energy or Urbach energy (E_u_), interpreted as the width of the bands localized states in the band gap, is an important parameter to characterize the disorder in the complex compound^31^. The absorption edge is found to be exponentially dependent on the incident photon energy and obeys the empirical Urbach rule Equation^32^: ${\rm{\alpha }}={{\rm{\alpha }}}_{0}\,\exp \,(\frac{{\rm{h}}{\rm{\gamma }}}{{{\rm{E}}}_{{\rm{u}}}})\,$, where α_0_ is a constant and E_u_ is the Urbach energy. Thus, by plotting Ln(α) as a function of energy hυ **[**Fig. S3**]**, the value of E_u_ is found to be 0.22 eV.

The spectral behavior of the real part of the refractive index n(E) of the complex compound was determined from reflectance and transmittance data using the following equation^33^. ${\boldsymbol{n}}=\frac{(1+{\boldsymbol{Ras}})}{(1-{\boldsymbol{Ras}})}+\sqrt{\frac{4{\boldsymbol{Ras}}}{{(1-{\boldsymbol{Ras}})}^{2}}-{{\boldsymbol{k}}}^{2}}$**;** Where ${{\boldsymbol{R}}}_{{\boldsymbol{as}}}=\frac{[\,2+{{\boldsymbol{T}}}^{2}\mbox{--}{(1-{\boldsymbol{R}})}^{2}]-\sqrt{{[2+{{\boldsymbol{T}}}^{2}-{(1-{\boldsymbol{R}})}^{2}]}^{2}-4(2-{\boldsymbol{R}}){\boldsymbol{R}}}}{2(2-{\boldsymbol{R}})}$ and ${\boldsymbol{k}}=\frac{-{\boldsymbol{\lambda }}}{4{\boldsymbol{\pi }}{\boldsymbol{t}}}\,\mathrm{Ln}(\frac{[{{\boldsymbol{T}}}^{2}-{(1-{\boldsymbol{R}})}^{2}]+\sqrt{{[{{\boldsymbol{T}}}^{2}-{(1-{\boldsymbol{R}})}^{2}]}^{2}+4{{\boldsymbol{T}}}^{2}]}}{2{\boldsymbol{T}}})$, R is the reflectance, T is the transmittance, k is the extinction coefficient and t is the thickness of the slab (t = 1 mm).

**Figure 5:**
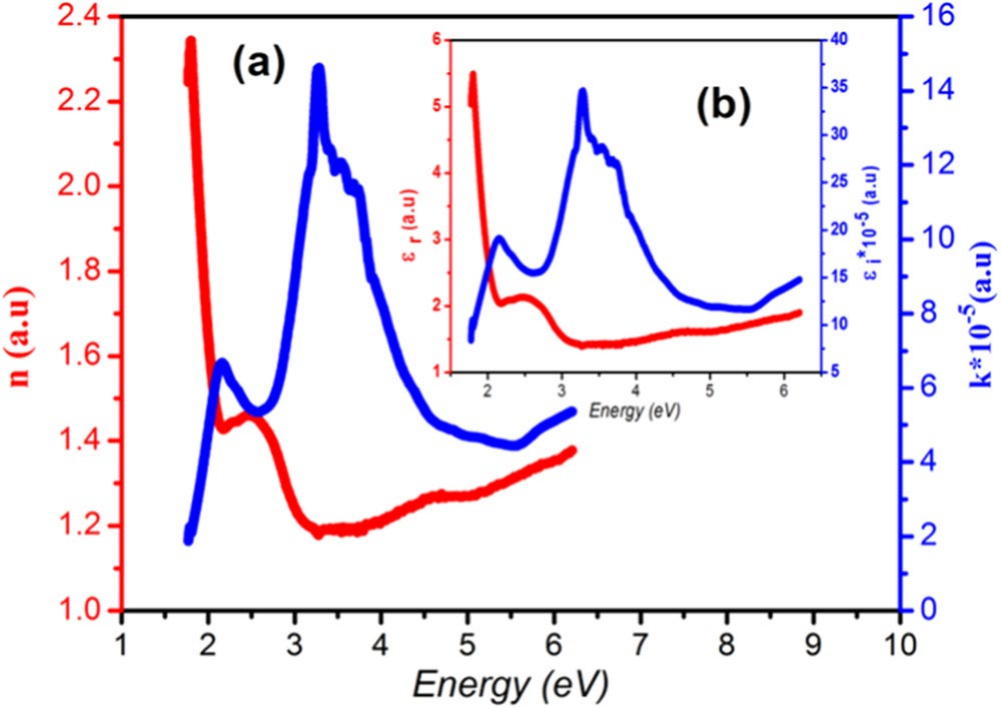
(**a**) Refractive index dispersion (n) and extinction coefficient (k) versus energy of (C_6_H_10_N_2_)_2_[Co(H_2_O)_4_P_2_Mo_5_O_23_].6H_2_O. (**b**) Real part (ε_r_) and imaginary part (ε_i_) of the dielectric permittivity versus energy of (C_6_H_10_N_2_)_2_[Co(H_2_O)_4_P_2_Mo_5_O_23_].6H_2_O.

The variation of the real refractive index n as well as the extinction coefficient k with the energy in the range of 1.7 to 6 eV are illustrated in Fig. 5a. The refractive index shows a large dispersive behavior on the visible region. Contrariwise, at the UV part, the compound can be assumed as non dispersive, having a constant refractive value (n_∞_ = 1.41). The observed variation of the refractive index with energy is exclusively due to polarizability changes and the anomalous dispersion may be correspond to the photon energy of the forbidden gaps energy that the compound holds^34^. The maximum of refractive index reached to 2.35 at 1.81 eV, this large value of “n” known in the visible region of electromagnetic spectrum reveals that the sample can become a promising candidate for visible optical communication devices. The imaginary part of the refractive index k, denotes absorption of optical energy by the semiconductor. In the spectral regions where the processes of absorption are weak or absent, as in the case of the sub band gap range, k is minimal, whereas in regions of strong absorption, the magnitude of k is large.

The real and imaginary parts of dielectric constant are calculated using the relation^35^: ε_r_ = n^2^− k^2^ and ε_i_* = *2nk. Where ε_0_ is the dielectric constant in the absence of any contribution from free carriers. The real part (ε_r_) of the dielectric constant of the complex indicates the extent to which the velocity of light is reduced, while the imaginary part (ε_i_) indicates the energy absorption by the complex^36^. The variation of ε_r_ and ε_i_ with the energy are shown in Fig. 5b, respectively. The comparison between k(λ) and ε_i_ (λ) follow closely each other in the complex and the low values of ε_i_ infer that the optical loss due to absorption is very weak. Therefore, the ratio between ε_i_ and ε_r_ gives information about the loss factor^37^. The ε_r_ (E) curve presents a similar behavior of n (E) plot because of the smaller values of k^2^.

**Figure 6:**
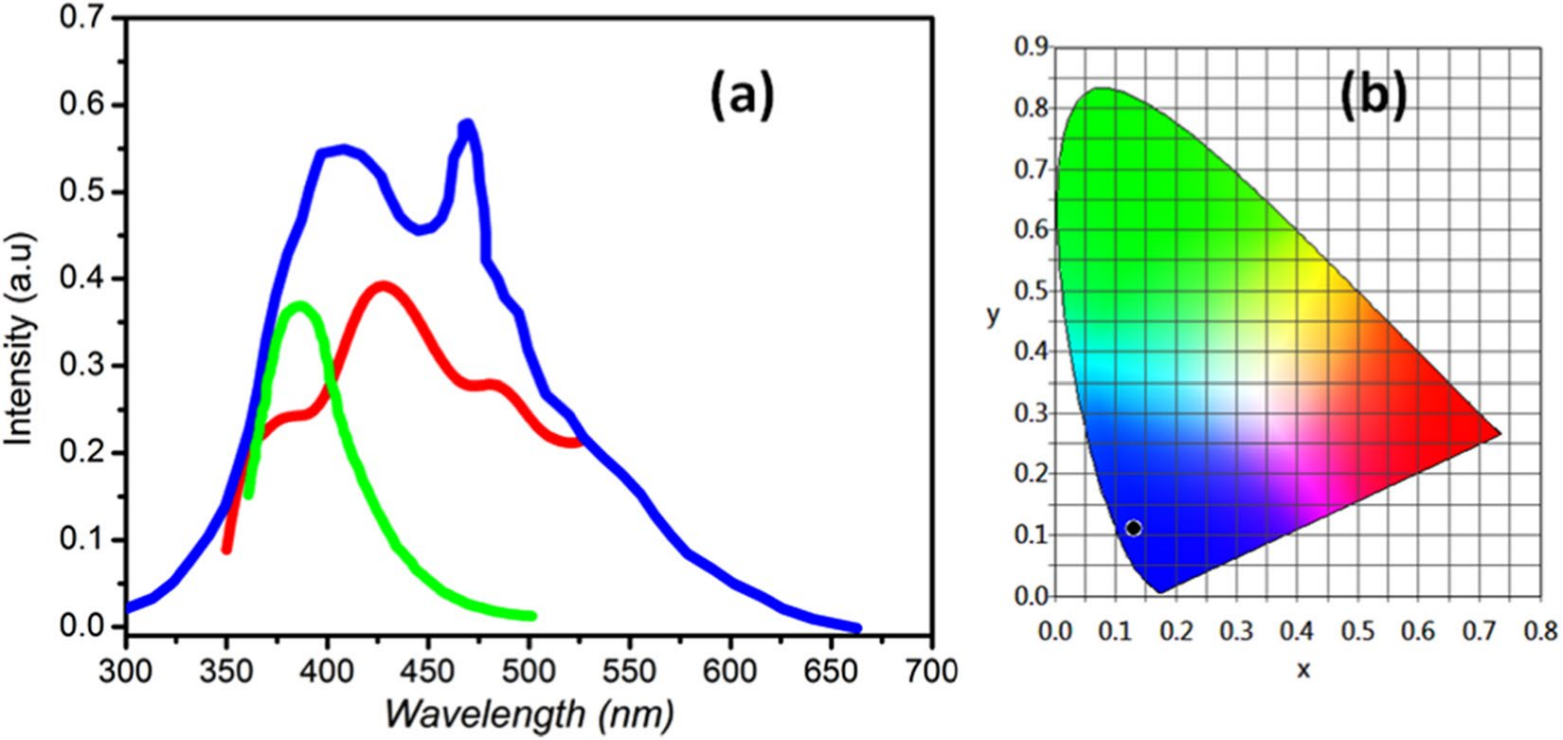
(**a**) The solid-state fluorescent emission spectra of the 3-picolylamine (Green), P_2_Mo_5_ (Bleu) and (C_6_H_10_N_2_)_2_[Co(H_2_O)_4_P_2_Mo_5_O_23_].6H_2_O (Red); (**b**) The CIE color coordinate diagrams of C_6_H_10_N_2_)_2_[Co(H_2_O)_4_P_2_Mo_5_O_23_].6H_2_O.

The solid state fluorescence properties of the complex compound are investigated at room temperature. As expected in Fig. 6a, upon photoexcitation at 350 nm, the title compound shows three main emission peaks in the visible region at 370, 402 and 461 nm. To understand the origin of these emission peaks, the emission spectra of the free P_2_Mo_5_ cluster and 3-picolylamine has been compared. Thus, the 3-picolylamine shows a broad emission at 378 nm, while the free P_2_Mo_5_ cluster display two main peaks at 408 and 470 nm upon excitation at 350 nm. Therefore, the luminescence emission peaks observed in the emission spectrun of the complex can be attributed to the π* → π transitions of the picolylammonium groups^38^ and the LMCT (O → Mo) of the polyanions^39^, respectively. However, the obvious decrease of the emission intensity of the complex, indicate that the coordination of P_2_Mo_5_ to Co^2+^ changes slightly the energy levels of the Strandberg cluster and affects their emission intensities^40^. The chromaticity diagram CIE 1931, upon excitation at 350 nm, of the simple is shown in Fig. 6b, the CIE colour coordinate (x, y) of the fluorescent emission peaks are (0.13, 0.11), illustrates that (C_6_H_10_N_2_)_2_[Co(H_2_O)_4_P_2_Mo_5_O_23_].6H_2_O belongs to a blue luminescent compounds. From the above results, it is concluded that the obtained compound is suitable for blue display device.

As concerns the infrared spectroscopy, the IR spectrum of the complex compound shown in Fig. S4, displays strong absorption bands at 907, 680 and 565 cm ^−1^ attributed to γ (Mo-Ot), γ (Mo-O-μ) and γ (Mo-O-Mo), respectively. The peaks at 1045 and 1099 cm^−1^ are attributed to the γ (P-O)^41^. While, 3- picolylamine shows vibration bands at 3110, 1570 and 1327 cm^−1^, assigned to ν(N-H), δ (N-H) and δ (C – N) groups, respectively^39^. The intense band in the range of 1646 cm^−1^ can be assigned to δ(O – H) and the broad and strong band at about 3360 cm^−1^ is associated to the water of crystallization.

### Magnetic properties

**Figure 7:**
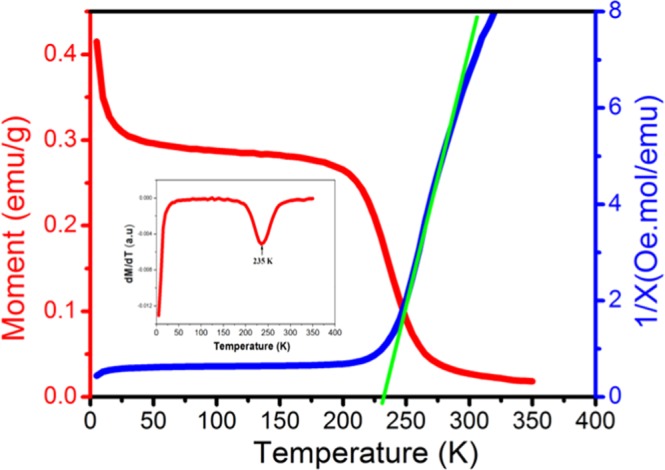
(**a**) Variation of the magnetization vs. Temperature for (C_6_H_10_N_2_)_2_[Co(H_2_O)_4_P_2_Mo_5_O_23_].6H_2_O (red). Temperature dependence of the inverse susceptibility for (C_6_H_10_N_2_)_2_[Co(H_2_O)_4_P_2_Mo_5_O_23_].6H_2_O sample (The solid line is the fitting result following the Curie-Weiss law) (blue).The plot of dM/dT curve as a function of temperature (inset).

Magnetic measurements have been performed on powder sample of (C_6_H_10_N_2_)_2_[Co(H_2_O)_4_P_2_Mo_5_O_23_].6H_2_O in temperature range from 2 to 300 K under a magnetic field of 500 Oe. As known, the valence states of Mo atoms are + 6 which indicate that they have no contribution to magnetism of compound and the magnetic character of the materials would reside in the Co(II) sites. Figure 7 shows that there is an abrupt PM - FM phase transition in the magnetization versus temperature (curve red) at T_C_ = 232 K which is determined by the minimum of the temperature derivative of the magnetization curve [Fig. 7 inset]. To analyze the magnetic phase transition in detail, we have also calculated the temperature dependence of the inverse susceptibility, χ^−1^ curve. As depicted in the blue curve Fig. 7, the shape of the curve between 300 and 225 K indicates the presence of a ferromagnetic exchange coupling between the neighboring Co^2+^ ions which is expected also from the structure data. Between 225 and 10 K, the χ^−1^ curve is almost constant and it rapidly falls towards zero at 4 K. The Curie -Weiss temperature θ_cw_ can be obtained by a linear regression of the paramagnetic region of the Curie -Weiss law [χ = C/(T − θ_cw_)]. Here, C and θ_cw_ are the Curie constant and the Curie-Weiss temperature, respectively. From C value, the experimental effective paramagnetic moment can be calculated as: $C=\frac{{N}_{A}}{3{K}_{B}}{\mu }_{eff}^{2}$, where N_A_ = 6.023.10^23^ mol^−1^ is the number of Avogadro, ${\mu }_{B}$ = 9.274.10^−21^ emu is the Bohr magneton and K_B_ = 1.38016.10^−23^ J.K^−1^ is the Boltzmann constant^42^. The spectroscopic splitting factor g can be deduced as g = $\frac{{\mu }_{eff}}{\sqrt{S(S+1)}}$, where S = 3/2 is the spin of the Co^2+^ ions. The exchange coupling constant $\frac{J}{{k}_{B}}$ and their associated effective field H_eff_, are calculated as: $\frac{J}{{k}_{B}}=\frac{3{\theta }_{cw}}{2ZS(S+1)}$ and ${H}_{eff}=\frac{2ZJS}{g\,{\mu }_{B}}$, respectively^43^. Z = 2 is the number of cobalt ions coupled to each cobalt ions.

The values of *C*, *θ*_*cw*_, ${\mu }_{eff}^{exp}$, g, J and H_eff_ for the applied magnetic field are tabulated in Table 2.

**Table 2:** Values of C, θ_cw_ and ${{\rm{\mu }}}_{{\rm{eff}}}^{\exp }$ for the applied magnetic field of (C_6_H_10_N_2_)_2_[Co(H_2_O)_4_P_2_Mo_5_O_23_].6H_2_O.

μ_0_H(Oe)	C (emu.K/mol)	θ_cw_ (K)	${{\boldsymbol{\mu }}}_{{\boldsymbol{e}}{\boldsymbol{f}}{\boldsymbol{f}}}^{{\boldsymbol{e}}{\boldsymbol{x}}{\boldsymbol{p}}}$ (μ_B_/Co)	g	J(K)	H_eff_ (Oe)
500	11.9	228.7	4.87	2.53	44	194

**Figure 8:**
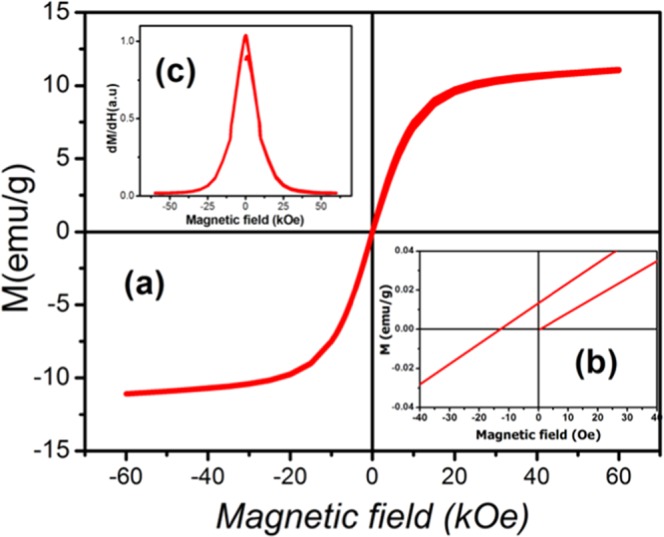
(**a**) Magnetic hysteresis (M–μ_0_H) loops of (C_6_H_10_N_2_)_2_[Co(H_2_O)_4_P_2_Mo_5_O_23_].6H_2_O. (**b**) Remnant magnetization and a coercive field. (**c**) dM/dH vs H plots recorded at 2 K.

The positive sign of the Curie-Weiss constant approves the ferromagnetic interactions between Co(II) centres and the large magnitude of J indicates a rather high ferromagnetic interaction between neighboring Co^2+^ centers. This result improved the capability of Strandberg-type polyoxoanion to mediate ferromagnetic interactions between metal atoms. One notices that for our ferromagnet Tc ~ θ_cw_ = N_w_C. According to this relation the magnitude of the Weiss constant, from the experimental value of T_C_ and C is N_w_ = 19 a.u. To investigate other magnetic properties, isothermal measurements were performed at 2 K. The results indicate that (C_6_H_10_N_2_)_2_[Co(H_2_O)_4_P_2_Mo_5_O_23_].6H_2_O compound possesses a reversible magnetic behavior without any evidence of hysteretic loops which represent a great advantage for efficient magnetic cooling [Fig. 8a]. The intercept of the linear fit to the high field data with the ordinate-axis gives a saturation magnetization for the magnetically ordered structure. We calculate M_sat_ = 11 emu/g, which corresponds to a saturation magnetic moment value ${{\rm{\mu }}}_{{\rm{S}}}^{\exp }\,$of 5.1 μ_B_/fu (2,55 μ_B_/Co^II^) which is slightly larger than the spin value expected for two uncoupled high-spin Co(II) ions (${\mu }_{S}^{th}$ = 3 μ_B_/Co^II^). On the other hand, field dependent magnetization reveals a hysteresis curve typical of a soft magnetic state, with a remnant magnetization of 0.013 emu/g and a coercive field of 13 Oe **[**Fig. 8b**]**. From the $\frac{{\rm{dM}}}{{\rm{dH}}}$ vs H plots at 2 K of the simple it is clearly to see only one peak which is suggested the interaction between the chains in studied material is very easy to overcome **[**Fig. 8c**]**.

### Theoretical investigation on electronic and magnetic structures

**Figure 9:**
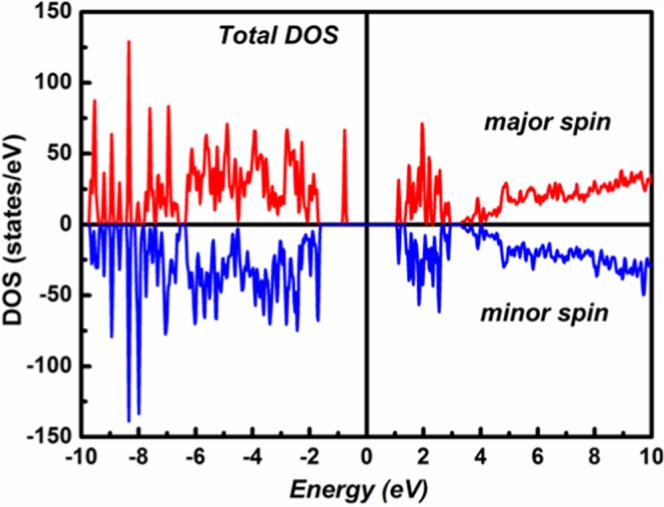
Total DOS of (C_6_H_10_N_2_)_2_[Co(H_2_O)_4_P_2_Mo_5_O_23_].6H_2_O complex from FLAPW calculations.

The Total Density of State (DOS) of (C_6_H_10_N_2_)_2_[Co(H_2_O)_4_P_2_Mo_5_O_23_].6H_2_O complex deduced from the band structure calculation is reported in Fig. 9. Here, the Fermi level is taken as reference. As seen, this DOS is not symmetrical with respect to energy axis, pointing out that the system is ferromagnetically ordered. Additionally, the total density of states has different band gaps in up spin channel (1.8 eV) and down spin channel (2.7 eV) which confirms the existence of a ferromagnetic semiconducting behavior^44^, a promising candidate among spintronic materials. Moreover, the obtained computational band gaps values are absolutely in accordance with our experimental optical values observed by the plot of $\frac{{\rm{d}}\,\mathrm{Ln}\,[{\rm{F}}({\rm{R}}){\rm{h}}{\rm{\gamma }}]}{{\rm{d}}\,({\rm{h}}{\rm{\gamma }})}$ versus energy.

**Figure 10:**
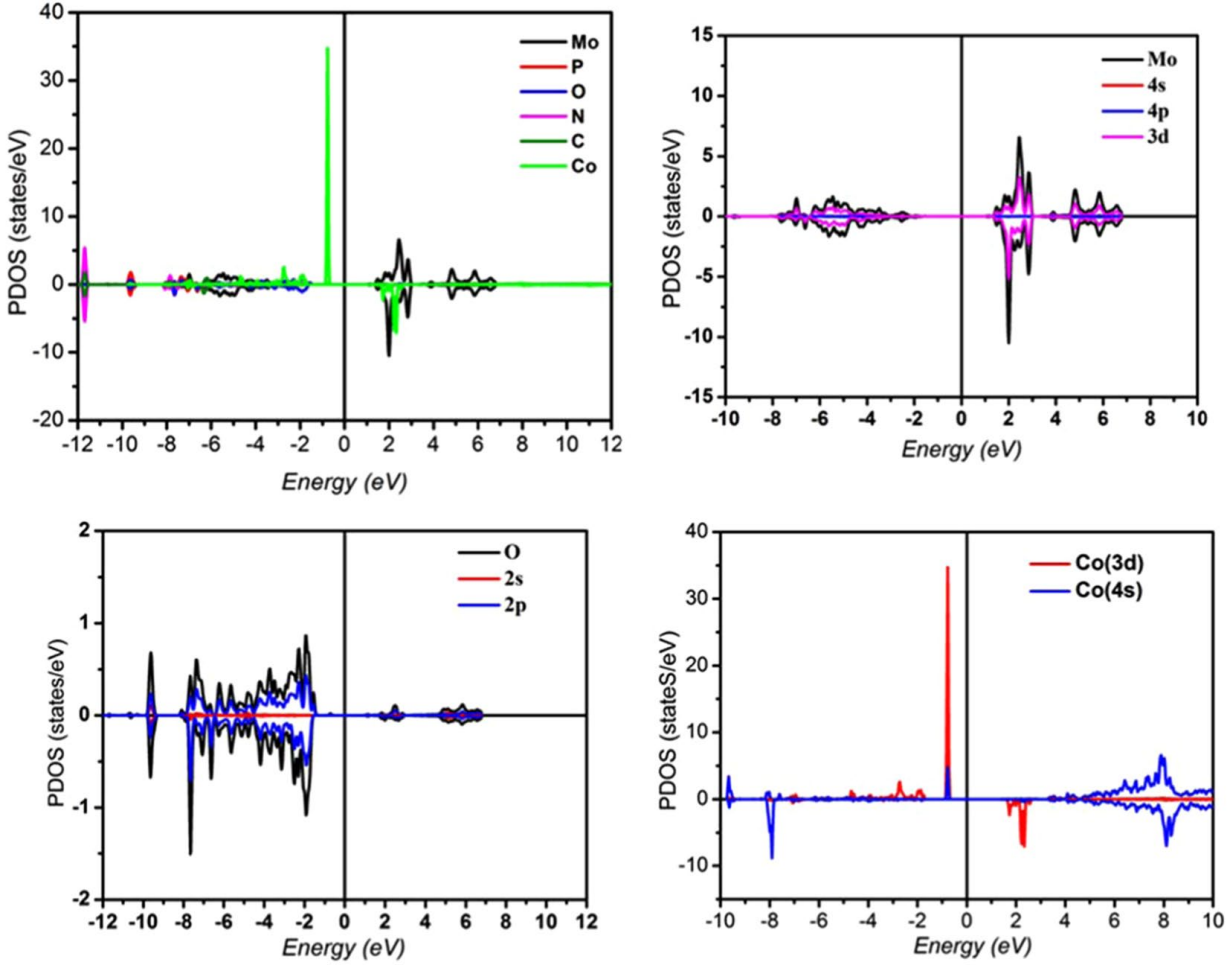
The l-decomposed DOS of Mo, P, O, N, C and Co atom in (C_6_H_10_N_2_)_2_[Co(H_2_O)_4_P_2_Mo_5_O_23_].6H_2_O from FLAPW calculations.

The detailed electronic structure was studied also from partial density of states (PDOS) plots as depicted in Fig. 10.

The PDOS revealed that the spin up of the valence band maximum (VBM) is mainly contributed by the hybridization between the spin-up parts of Co(3d) and O(2p) electrons while the spin down parts mainly consists of O(2p) electrons. The spin up of the conduction band minimum (CBM) mostly comprises of spin up parts of Mo(3d) states whereas the spin-down channel is contributed by the spin-down parts of Co(3d) and the spin down parts of Mo(3d) states which strongly hybridize with each other. Thus, the expected reason for the ferromagnetic semiconducting behavior in the simple may be due to the appreciable exchange splitting between the up and down spin channels of Co(3d) states as well as the hybridization of Co(3d) – Mo(3d) states which is more pronounced in down spin channel than in up spin channel.

In the present work the l-decomposed DOS of Co atoms have been studied in particular, only small contributions from Co(4 s) is revealed. It should be noticed that no contributions from Co(4p) is observed since is fully full and shifts to lower energies and behaves as core level. In addition, the Co oxidation state is calculated and found equal 2.5. Magnetic moment carried by Co atoms is computed as well and found equal 2.54 μ_B_, leading to a magnetic moment per formula of 5.08 μ_B_ which is in good agreement with our measured magnetization 5.1 μ_B_/f.u. In this context, we underline that, on the one hand, no magnetic moment is revealed on all atoms except Co atoms included in our compound.

On the other hand, we observe a transition from ferromagnetic state to paramagnetic state taking place at Curie temperature T_C_ = 232 K from our experimental measurements. Consequently, it should be concluded that a magnetic long-range order is revealed between neighboring Co cations. Considering the large Co-Co distance excising 9.5 Å, this magnetic interaction type is a characteristic of a magnetic super-exchange between Co(II) centers. This observed magnetic behavior is in accordance with early reported investigations on similar magnetic compounds^45,46^.

**Figure 11:**
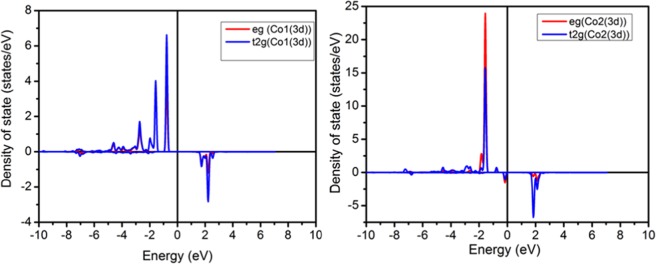
The calculated density of states of (C_6_H_10_N_2_)_2_[Co(H_2_O)_4_P_2_Mo_5_O_23_].6H_2_O in a ferromagnetic configuration from FLAPW calculations.

The orbital decomposed d-band density of state of Co(II) ions are depected in Fig. 11. The analysis of the PDOS suggest that the octahedral symmetry around Co atoms splits the Co (II) 3d levels into a triply degenerate t_2g_ band and a doubly degenerate e_g_ band. the five -fold 3d of both Co cations show that ${{\rm{t}}}_{2{\rm{g}}}^{\uparrow }$ and ${{\rm{e}}}_{{\rm{g}}}^{\uparrow }$ levels are completely occupied while the ${{\rm{t}}}_{2{\rm{g}}}^{\downarrow }$ and ${{\rm{e}}}_{{\rm{g}}}^{\downarrow }$ are partielly occupied. Such electronic configuration leading to a high spin state (HS) for Co cations. More importantly and according to the coupling model consider by B. Belhadji and al^47^, the obtained PDOS exhibits a competition between a ferromagnetic super-exchange, arising from the hybridization of the occupied ${{\rm{e}}}_{{\rm{g}}}^{\downarrow }$ states with the empty ${{\rm{t}}}_{2{\rm{g}}}^{\downarrow }$ states and an antiferromagnetic super-exchange due to the hybridization of the occupied ${{\rm{t}}}_{2{\rm{g}}}^{\uparrow }$ states with the empty ${{\rm{t}}}_{2{\rm{g}}}^{\downarrow }$ states. To distinguish the macroscopic dominant magnetic interaction a comparison between the experimental results and the computational results is performed. Thus, as the magnetic measurement present a dominant ferromagnetic ordering in the simple consequencly the ferromagnetic super-exchange occuring from (occupied ${{\rm{e}}}_{{\rm{g}}}^{\downarrow }$ - empty ${{\rm{t}}}_{2{\rm{g}}}^{\downarrow }$) 3d orbitals is stronger than the antiferromagnetic super-exchange occuring from occupied (${{\rm{t}}}_{2{\rm{g}}}^{\uparrow }$ – empty ${{\rm{t}}}_{2{\rm{g}}}^{\downarrow }$) 3d orbitals of Co atoms.

## Conclusion

In summary, we successfully synthesized a novel Strandberg-type hybrid complex based on the {P_2_Mo_5_} anions bridged by cobalt cations generating 1D zig zag chain structure. A network of hydrogen bonds, between the organic and inorganic components in the crystal, leads to a three-dimensional supramolecular architecture. The analysis of the DRS data as well as the total DOS calculation reveals that this polymer can be identified as a ferromagnetic semiconductor. The study of the luminescent properties at room temperature demonstrated that the obtained material is a blue emission compound. Refractive index and dielectric results reveals that the elaborated semiconductor can be used as a candidate for visible light device. Field dependent magnetization presents a hysteresis curve typical of a soft magnetic state as well electronic structure calculation and temperature dependent magnetization measurement confirms the presence of long-range ferromagnetic order between Co cations, this magnetic interaction type is a characteristic of a magnetic super-exchange. At the end, our experiments and theoretical analyses both verify the coexistence of ferromagnetism and semiconducting conductivity in the studied polymer, which makes (C_6_H_10_N_2_)_2_[Co(H_2_O)_4_P_2_Mo_5_O_23_].6H_2_O to be a vital candidate in novel spintronics.

## Supplementary information

Supplementary information.
