# Supplementary material for: Structure, optical and magnetic properties of a novel homometallic coordination polymers: Experimental and Computational studies
Source: Sci Rep. 2020 Jan 28;10:1316. doi: 10.1038/s41598-020-58176-3 (PMC6987158; doi:10.1038/s41598-020-58176-3)
Supplement: Supplementary file 1 — Supplementary information. [file 41598_2020_58176_MOESM1_ESM.docx]

# Structure, optical and magnetic properties of a novel homometallic coordination polymers: Experimental and Computational studies

Y. Ammari*^a^, N. Baaalla^c^, E.K. Hlil^b^, S. Abid^a^

*^a^ Université de Carthage, Faculté des sciences de Bizerte, LR13ES08 Laboratoire de chimie des matériaux, 7021, Zarzouna Bizerte, Tunisie*

*^b^ Institut Néel, CNRS, Université Grenoble Alpes, 25 rue des Martyrs BP 166 38042 Grenoble cedex 9, France.*

*^c^ LaMCScI, Laboratory of Condensed Matter and Interdisciplinary Sciences, B.P. 1014, Faculty of Science,  Mohammed V University , Rabat, Morocco.*

**Table S1.** Hirshfeld contact surfaces and derived random contact and enrichment ratios for

(C_6_H_10_N_2_)_2_[Co(H_2_O)_4_P_2_Mo_5_O_23_].6H_2_O

|  |  | H | C | O | N | Co |
| --- | --- | --- | --- | --- | --- | --- |
| Contacts (%) | H | 31.8 | *** | *** | *** | *** |
|  | C | 3.1 | 0.6 | *** | *** | *** |
|  | O | 52.2 | 5 | 1.7 | *** | *** |
|  | N | 0.9 | 0 | 0.6 | 0 | *** |
|  | Co | 0.2 | 0 | 3.9 | 0 | 0 |
| Surface (%) |  | 60 | 4.65 | 32.55 | 0.75 | 2.05 |
| Random  (%) | H | 36 | *** | *** | *** | *** |
|  | C | 5.58 | 0.21 | *** | *** | *** |
|  | O | 39.06 | 3.02 | 10.59 | *** | *** |
|  | N | 0.9 | 0.069 | 0.48 | 0.005 | *** |
|  | Co | 2.46 | 0.19 | 1.33 | 0.03 | 0.04 |
| Enrichment | H | 0.88 | *** | *** | *** | *** |
|  | C | 0.55 | *** | *** | *** | *** |
|  | O | 1.33 | 1.65 | 0.16 | *** | *** |
|  | N | 1 | 0 | 1.22 | 0 | *** |
|  | Co | *** | 0 | 2.92 | 0 | 0 |

**Table S2.** Selected Bond Lengths (Å), magnitude of distortion (Δd) of MoO_6_ octahedral and Bond Angles (°) for the complex Compound (C_6_H_10_N_2_)_2_[Co(H_2_O)_4_P_2_Mo_5_O_23_].6H_2_O

| Mo1—O14 | 1.696 (2) | Mo2—O22 | 1.695 (3) | Mo3—O21 | 1.695 (2) |
| --- | --- | --- | --- | --- | --- |
| Mo1—O15 | 1.709 (2) | Mo2—O23 | 1.706 (3) | Mo3—O20 | 1.727 (3) |
| Mo1—O13 | 1.903 (2) | Mo2—O12 | 1.939 (2) | Mo3—O11 | 1.911 (2) |
| Mo1—O9 | 1.922 (3) | Mo2—O13 | 1.940 (2) | Mo3—O12 | 1.924 (2) |
| Mo1—O4 | 2.309 (2) | Mo2—O8 | 2.211 (2) | Mo3—O3 | 2.231 (2) |
| Mo1—O7 | 2.314 (2) | Mo2—O4 | 2.349 (2) | Mo3—O8 | 2.388 (2) |
| **Δd = 1.265** | | **Δd= 1.233** | | **Δd= 1.232** | |

| Mo4—O18 | 1.702 (3) | Mo5—O17 | 1.701 (3) | Co1—O1i | 2.056 (2) |
| --- | --- | --- | --- | --- | --- |
| Mo4—O19 | 1.707 (3) | Mo5—O16 | 1.712 (2) | Co1—O1 | 2.056 (2) |
| Mo4—O11 | 1.911 (2) | Mo5—O10 | 1.917 (2) | Co1—OW2 | 2.094 (2) |
| Mo4—O10 | 1.939 (2) | Mo5—O9 | 1.950 (2) | Co1—OW2i | 2.094 (2) |
| Mo4—O6 | 2.242 (2) | Mo5—O2 | 2.189 (2) | Co1—OW1i | 2.115 (3) |
| Mo4—O2 | 2.311 (2) | Mo5—O7 | 2.390 (2) | Co1—OW1 | 2.115 (3) |
| **Δd = 1.192** | | **Δd = 1.265** | | **Δd = 0** | |

| Co2—O5ii | 2.047 (2) | P1—O1 | 1.512 (3) | P2—O5 | 1.517 (3) |
| --- | --- | --- | --- | --- | --- |
| Co2—O5 | 2.047 (2) | P1—O3 | 1.520 (2) | P2—O6 | 1.520 (2) |
| Co2—OW4 | 2.116 (3) | P1—O2 | 1.551 (2) | P2—O8 | 1.554 (2) |
| Co2—OW4ii | 2.116 (3) | P1—O4 | 1.552 (2) | P2—O7 | 1.560 (2) |
| Co2—OW3 | 2.127 (3) |  |  |  |  |
| Co2—OW3ii | 2.127 (3) |  |  |  |  |
| **Δd = 0** | |  |  |  |  |

| N1—C1 | 1.331 (5) | N3—C11 | 1.323 (5) |
| --- | --- | --- | --- |
| N1—C5 | 1.333 (6) | N3—C7 | 1.332 (5) |
| N4—C12 | 1.482 (6) | N2—C6 | 1.489 (5) |
| C2—C1 | 1.375 (5) | C2—C3 | 1.378 (6) |
| C6—C2 | 1.499 (5) | C8—C9 | 1.379 (6) |
| C5—C4 | 1.360 (6) | C8—C7 | 1.385 (5) |
| C10—C11 | 1.369 (6) | C8—C12 | 1.514 (6) |
| C10—C9 | 1.380 (6) | C3—C4 | 1.377 (6) |

| O14—Mo1—O15 | 103.26 (12) | O1—Co1—OW2i | 90.60 (10) |
| --- | --- | --- | --- |
| O14—Mo1—O13 | 100.44 (12) | O1i—Co1—O2Wi | 89.40 (10) |
| O15—Mo1—O13 | 101.66 (12) | OW2—Co1—OW2i | 180.0 |
| O14—Mo1—O9 | 100.54 (12) | O1—Co1—OW1i | 86.17 (10) |
| O7—Mo1—O9 | 97.97 (12) | O1i—Co1—OW1i | 93.83 (10) |
| O13—Mo1—O9 | 146.99 (9) | OW2—Co1—OW1i | 89.84 (11) |
| O14—Mo1—O3 | 167.34 (10) | OW2i—Co1—OW1i | 90.16 (11) |
| O15—Mo1—O3 | 89.03 (10) | O1—Co1—OW1 | 93.83 (10) |
| O13—Mo1—O3 | 73.64 (9) | O1i—Co1—OW1 | 86.17 (10) |
| O9—Mo1—O3 | 80.48 (9) | OW2—Co1—OW1 | 90.16 (11) |
| O14—Mo1—O7 | 87.39 (10) | OW2i—Co1—OW1 | 89.84 (11) |
| O15—Mo1—O7 | 167.97 (10) | OW1i—Co1—OW1 | 180.0 |
| O13—Mo1—O7 | 81.55 (9) | O5ii—Co2—O5 | 180.000 (1) |
| O9—Mo1—O7 | 74.26 (9) | O5ii—Co2—OW4ii | 92.17 (10) |
| O3—Mo1—O7 | 80.71 (8) | O5—Co2—OW4ii | 87.83 (10) |
| O22—Mo2—O23 | 104.34 (14) | O5ii—Co2—OW4 | 87.83 (10) |
| O22—Mo2—O12 | 97.37 (11) | O5—Co2—OW4 | 92.17 (10) |
| O23—Mo2—O12 | 99.07 (12) | OW4ii—Co2—OW4 | 180.000 (1) |
| O22—Mo2—O13 | 101.17 (11) | O5ii—Co2—OW3 | 88.74 (9) |
| O23—Mo2—O13 | 95.78 (11) | O5—Co2—OW3 | 91.26 (9) |
| O12—Mo2—O13 | 152.54 (10) | OW4ii—Co2—OW3 | 96.10 (11) |
| O22—Mo2—O8 | 157.03 (12) | OW4—Co2—OW3 | 83.90 (11) |
| O23—Mo2—O8 | 97.79 (11) | O5ii—Co2—OW3ii | 91.26 (9) |
| O12—Mo2—O8 | 72.82 (9) | O5—Co2—OW3ii | 88.74 (9) |
| O13—Mo2—O8 | 82.40 (9) | OW4ii—Co2—OW3ii | 83.90 (11) |
| O22—Mo2—O3 | 86.04 (12) | OW4—Co2—OW3ii | 96.10 (11) |
| O23—Mo2—O3 | 165.59 (11) | OW3—Co2—OW3ii | 180.000 (1) |
| O12—Mo2—O3 | 89.30 (9) | O1—P1—OW2 | 112.15 (13) |
| O13—Mo2—O3 | 72.09 (9) | O1—P1—O2 | 108.41 (14) |
| O8—Mo2—O3 | 73.37 (8) | O2—P1—O2 | 106.86 (13) |
| O18—Mo4—O19 | 101.64 (14) | O1—P1—O1 | 110.79 (14) |
| O18—Mo4—O11 | 101.25 (12) | O2—P1—O1 | 109.12 (14) |
| O19—Mo4—O11 | 100.32 (12) | O4—P1—O1 | 109.41 (13) |
| O18—Mo4—O10 | 100.98 (11) | O5—P2—O4 | 110.94 (14) |
| O19—Mo4—O10 | 99.18 (12) | O5—P2—OW4 | 109.50 (14) |
| O11—Mo4—O10 | 146.65 (10) | O6—P2—OW4 | 107.13 (12) |
| O18—Mo4—O6 | 84.86 (12) | O5—P2—OW3 | 110.51 (13) |
| O19—Mo4—O6 | 173.41 (11) | O6—P2—OW3 | 108.44 (14) |
| O11—Mo4—O6 | 79.25 (10) | O8—P2—OW3 | 110.27 (13) |
| O10—Mo4—O6 | 78.32 (10) | P1—O5—Mo1 | 126.78 (13) |
| O18—Mo4—O4 | 167.92 (11) | P1—O5—Mo2 | 128.41 (13) |
| O19—Mo4—O4 | 88.00 (11) | Mo1—O5—Mo2 | 92.26 (8) |
| O11—Mo4—O4 | 83.93 (9) | P2—O10—Mo4 | 124.84 (13) |
| O10—Mo4—O4 | 69.98 (9) | P1—O3—Co1 | 135.75 (15) |
| O6—Mo4—O4 | 85.41 (8) | P1—O4—Mo3 | 123.85 (13) |
| O17—Mo5—O16 | 104.74 (14) | P2—O7—Mo1 | 125.96 (13) |
| O17—Mo5—O10 | 100.49 (12) | P2—O7—Mo5 | 128.04 (13) |
| O16—Mo5—O10 | 99.40 (11) | Mo1—O7—Mo5 | 91.92 (8) |
| O17—Mo5—O9 | 94.61 (12) | P1—O4—Mo5 | 127.95 (13) |
| O16—Mo5—O9 | 97.32 (11) | P1—O4—Mo4 | 135.62 (13) |
| O10—Mo5—O9 | 153.76 (10) | Mo5—O4—Mo4 | 96.10 (8) |
| O17—Mo5—O4 | 96.99 (11) | P2—O8—Mo2 | 130.20 (13) |
| O16—Mo5—O4 | 158.05 (12) | P2—O8—Mo3 | 133.46 (13) |
| O10—Mo5—O4 | 73.17 (9) | Mo2—O8—Mo3 | 94.82 (8) |
| O9—Mo5—O4 | 83.84 (9) | P2—O5—Co2 | 126.70 (14) |
| O17—Mo5—O7 | 163.41 (11) | Mo3—O12—Mo2 | 122.54 (11) |
| O16—Mo5—O7 | 87.08 (11) | Mo3—O11—Mo4 | 148.71 (13) |
| O10—Mo5—O7 | 88.77 (10) | Mo1—O13—Mo2 | 121.83 (12) |
| O9—Mo5—O7 | 72.01 (9) | Mo1—O9—Mo5 | 121.74 (12) |
| O4—Mo5—O7 | 72.37 (8) | C11—N3—C7 | 123.0 (4) |
| O21—Mo3—O20 | 101.95 (12) | C9—C8—C7 | 117.7 (4) |
| O21—Mo3—O11 | 103.96 (12) | C9—C8—C12 | 122.8 (4) |
| O20—Mo3—O11 | 98.67 (12) | C7—C8—C12 | 119.4 (4) |
| O21—Mo3—O12 | 100.26 (11) | C1—N1—C5 | 123.1 (4) |
| O20—Mo3—O12 | 99.20 (12) | Mo5—O10—Mo4 | 120.51 (12) |
| O11—Mo3—O12 | 146.04 (10) | N2—C6—C2 | 112.9 (3) |
| O21—Mo3—O2 | 86.90 (11) | N1—C5—C4 | 118.8 (4) |
| O20—Mo3—O2 | 171.11 (10) | N3—C7—C8 | 120.1 (4) |
| O11—Mo3—O2 | 79.81 (10) | C11—C10—C9 | 119.4 (4) |
| O12—Mo3—O2 | 78.10 (10) | C1—C2—C3 | 117.8 (3) |
| O21—Mo3—O8 | 168.50 (10) | C1—C2—C6 | 119.4 (4) |
| O20—Mo3—O8 | 84.42 (10) | C3—C2—C6 | 122.6 (4) |
| O11—Mo3—O8 | 84.28 (9) | N3—C11—C10 | 119.3 (4) |
| O12—Mo3—O8 | 69.04 (8) | N4—C12—C8 | 112.1 (4) |
| O2—Mo3—O8 | 86.71 (8) | C4—C3—C2 | 120.3 (4) |
| O1—Co1—O1i | 180.00 (10) | N1—C1—C2 | 120.1 (4) |
| O1—Co1—OW2 | 89.40 (10) | C5—C4—C3 | 119.9 (4) |
| O1i—Co1—OW2 | 90.60 (10) | C8—C9—C10 | 120.3 (4) |

**Symmetry codes: (i) −*x*+1, −*y*+1, −*z*+1; (ii) −*x*, −*y*+1, −*z*+2.**

Table S3. Hydrogen-bond geometry (Å, deg.) of the title compound (C_6_H_10_N_2_)_2_[Co(H_2_O)_4_P_2_Mo_5_O_23_].6H_2_O

| Donor --- H....Acceptor | D – H (Å) | H...A (Å) | D...A (Å) | D - H...A(°) |
| --- | --- | --- | --- | --- |
| N(1)-H(1)... OW(3) | 0.86 | 2.36 | 2.996(4) | 131 |
| N(1)-H(1)... O(5) | 0.86 | 2.07 | 2.827(4) | 146 |
| N(2)-H(2A)... OW(7) | 0.89 | 1.91 | 2.779(5) | 164 |
| N(2)-H(2B)... O(20) | 0.89 | 2.52 | 2.989(4) | 113 |
| N(2)-H(2B)… O(21) | 0.89 | 2.04 | 2.865(4) | 154 |
| N(2)-H(2C)... O(6) | 0.89 | 2.10 | 2.852(4) | 141 |
| N(2)-H(2C)... O(11) | 0.89 | 2.54 | 3.335(4) | 149 |
| N(3)-H(3) ... O(1) | 0.86 | 1.95 | 2.734(5) | 151 |
| N(4)-H(45A) ... OW(5) | 0.89 | 2.05 | 2.919(6) | 164 |
| N(4)-H(45B) ... O(21) | 0.89 | 2.29 | 2.997(4) | 136 |
| N(4)-H(45B) ... OW(8) | 0.89 | 2.35 | 2.936(4) | 123 |
| N(4)-H(45C) ... O(16) | 0.89 | 2.12 | 2.941(4) | 152 |
| OW1-H(12) ... OW(9A) | 0.85 | 2.02 | 2.821(8) | 157 |
| OW2-H(13) ... OW(8) | 0.85 | 2.09 | 2.748(4) | 134 |
| OW2-H(14) ... O(9) | 0.85 | 2.02 | 2.847(4) | 163 |
| OW2-H(14) ... O(15) | 0.85 | 2.46 | 2.986(4) | 121 |
| OW5-H(15) ... O(15) | 0.85 | 2.03 | 2.879(4) | 175 |
| OW5-H(16) ... O(19) | 0.85 | 2.18 | 3.028(5) | 175 |
| OW8-H(17) ... O(3) | 0.85 | 2.05 | 2.747(4) | 138 |
| OW8-H(18) ... OW(9A) | 0.85 | 2.46 | 3.070(8) | 130 |
| OW3-H(23) ... O(7) | 0.85 | 2.18 | 2.913(3) | 144 |
| OW3-H(23) ... O(14) | 0.85 | 2.58 | 3.082(4) | 119 |
| OW3-H(24) ... OW(7) | 0.85 | 2.02 | 2.763(4) | 145 |
| OW4-H(25) … O(6) | 0.85 | 2.21 | 2.831(4) | 130 |
| OW4-H(26) … OW(6) | 0.85 | 2.35 | 2.689(5) | 104 |
| OW7-H(27) … OW(10B) | 0.85 | 2.02 | 2.858(12) | 170 |
| OW7-H(28) … OW(6) | 0.85 | 2.10 | 2.843(5) | 145 |
| OW6-H(30) … O(23) | 0.86 | 1.87 | 2.718(5) | 169 |
| OW6-H(31) … O(13) | 0.85 | 1.93 | 2.786(4) | 174 |
| OW1-H(111) … O(4) | 0.85 | 2.22 | 2.874(4) | 134 |
| OW1-H(111) … O(15) | 0.85 | 2.58 | 3.130(4) | 124 |
| OW1-H(111) … O(22) | 0.85 | 2.48 | 3.168(4) | 138 |
| OW10B-H(138)...O(18) | 0.86 | 2.56 | 2.845(8) | 101 |
| OW9A-H(236)... O(10) | 0.85 | 2.24 | 2.883(8) | 133 |
| OW9A-H(236)... O(17) | 0.85 | 2.39 | 3.133(8) | 146 |
| OW10B-H(238)...O(12) | 0.85 | 2.24 | 2.802(8) | 124 |
| OW10B-H(238)...O(21) | 0.85 | 2.35 | 3.160(8) | 160 |
| C(1)-H(1A)... O(20) | 0.93 | 2.43 | 3.220(5) | 143 |
| C(5)-H(5)... O(16) | 0.93 | 2.51 | 3.310(6) | 144 |
| C(7)-H(7)... O(17) | 0.93 | 2.54 | 3.204(6) | 129 |
| C(9)-H(9)... O(3) | 0.93 | 2.49 | 3.390(5) | 162 |
| C(9)-H(9)... O(21) | 0.93 | 2.51 | 3.112(6) | 123 |
| C(10)-H(10)... O(27) | 0.93 | 2.33 | 3.227(5) | 162 |
|  |  |  |  |  |


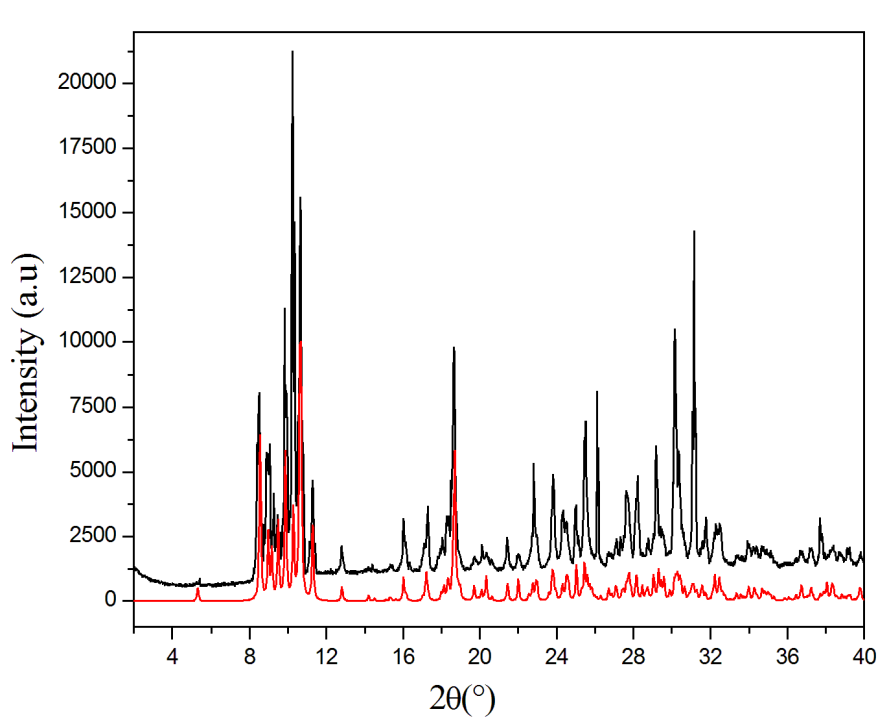


Figure S1 Simulated (a) and experimental (b) powder X-ray diffraction patterns of the simple.


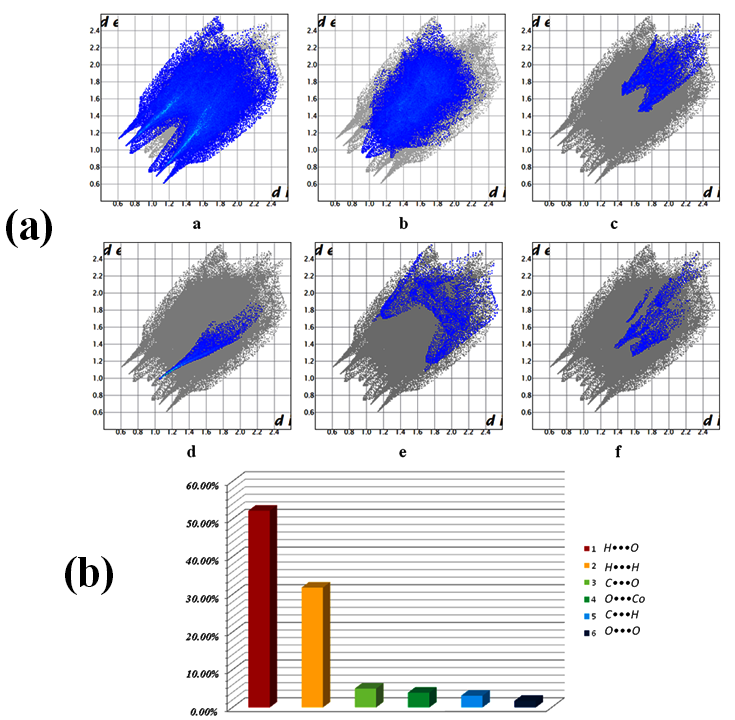


Figure S2 (a).Fingerprint plots: (a) H…O/O…H; (b) H…H; (c) C…O/O…C; (d) O…Co/Co…O; (e) C…H/ H…C ; (f) O…O. (b). Percentage contribution of all interactions around the Strandberg cluster.


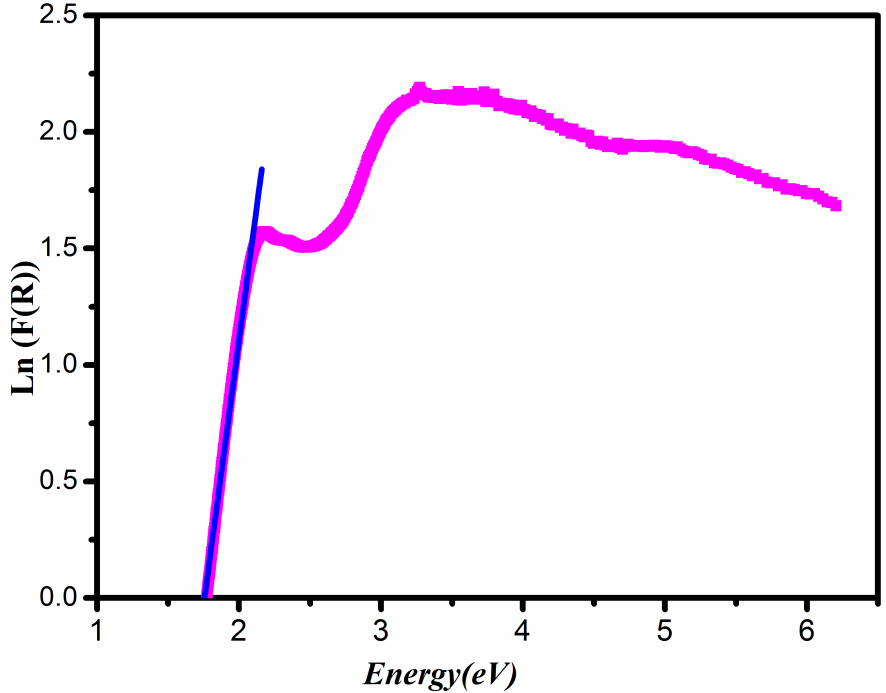


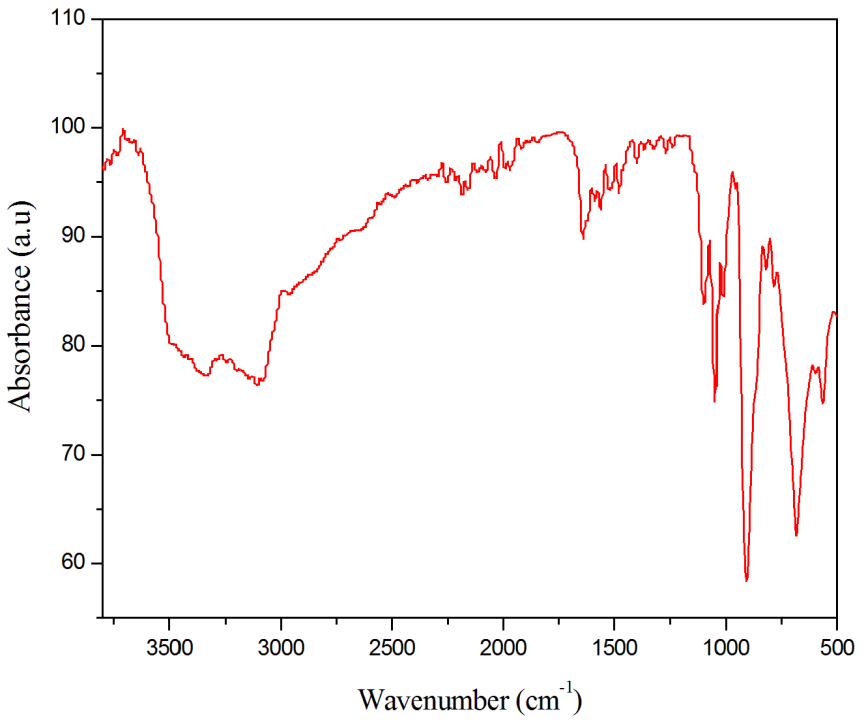


Figure S4 The IR spectrum of (C_6_H_10_N_2_)_2_[Co(H_2_O)_4_P_2_Mo_5_O_23_].6H_2_O.

Figure S3 The logarithmic variation of the absorption coefficient versus photon energy for (C_6_H_10_N_2_)_2_[Co(H_2_O)_4_P_2_Mo_5_O_23_].6H_2_O.
